# Sub-Lethal Effects of Bifenthrin and Imidacloprid on *Megacephala carolina carolina* L. (Coleoptera: Carabidae) in Turfgrass

**DOI:** 10.3390/insects14010008

**Published:** 2022-12-22

**Authors:** Shimat V. Joseph

**Affiliations:** Department of Entomology, University of Georgia, 1109 Experiment Street, Griffin, GA 30223, USA; svjoseph@uga.edu; Tel.: +1-770-228-7312

**Keywords:** tiger beetle, lawn, golf course, bifenthrin, imidacloprid, integrated pest management, nontarget effects

## Abstract

**Simple Summary:**

Tiger beetles are important predators in turfgrass in Georgia, USA. Many insecticides are sprayed on turfgrass to manage pests. Among them, bifenthrin and imidacloprid are the most commonly used insecticides in turfgrass. These insecticides may harm beneficial insects or the behavior of tiger beetles in turfgrass. Therefore, the objective was to determine whether lower doses of bifenthrin and imidacloprid would alter the normal hunting behavior of larvae and adult tiger beetles. Lower doses of bifenthrin altered the normal hunting behavior of larvae and adult tiger beetles. However, lower doses of imidacloprid did not alter the larval behavior. These results suggest that not all insecticides are equally harmful to the beneficial insects in turfgrass. Turfgrass managers should carefully inspect the presence of tiger beetle holes before spraying insecticides on turfgrass.

**Abstract:**

The tiger beetle, *Megacephala carolina carolina* L. (Coleoptera: Carabidae), is a common predator in turfgrass and ornamental landscapes in Georgia, USA. Among insecticides used in turfgrass to control foliar and root-feeding insect pests, bifenthrin and imidacloprid are routinely used. It was unclear whether sub-lethal doses of bifenthrin and imidacloprid could cause nontarget effects on larvae and *M. carolina carolina* adults. Thus, the objective was to determine the sub-lethal effects of bifenthrin and imidacloprid on larvae and *M. carolina carolina* adults. The results show that *M. carolina carolina* larvae actively hunt for passing prey by waiting at the hole of the tunnel during the day and nighttime. This larval behavior was affected by sub-lethal doses (up to 25% of full label rate) of bifenthrin but not of imidacloprid. The walking behavior of adult *M. carolina carolina* was also altered when exposed to sub-lethal doses of bifenthrin as they traveled further distances at greater velocities than the nontreated control. The results imply that turfgrass managers should avoid treating lawns where tiger beetles have actively colonized.

## 1. Introduction

Many insect pests infest turfgrass and cause serious damage [1]. Among turfgrass pests, fall armyworm, *Spodoptera frugiperda* (JE Smith), black cutworm, *Agrotis ipsilon* (Hufnagel) [2,3], mole crickets (tawny mole cricket, *Scapteriscus vicinus* Scudder, southern mole cricket, *Scapteriscus borellii* Giglio-Tos) [2], and two-lined spittlebug, *Prosapia bicincta* (Say) [4], stand out as the major pests that affect various turfgrass systems, such as golf courses, sod farms, and public and residential lawns. In the USA, turfgrass is a major agricultural commodity, contributing an estimated USD 35.1 billion in revenue annually to the economy [5]. In Georgia (USA), the value of sod production has been estimated at USD 118.3 million, and about 10,785 ha were under production spread across 64 counties in the state [6]. Turfgrass practitioners often rely on insecticides for pest management [2]. Pyrethroids and neonicotinoids are two frequently used insecticide classes by the turfgrass industry to manage many important arthropod pests. Pyrethroids are recommended for the management of many arthropod pests, such as fall armyworm, cutworm, southern chinch bug, *Blissus insularis* Barber, and many others [2,7]. Similarly, imidacloprid is recommended to manage many arthropod pests, such as billbugs, *Sphenophorous* spp., southern chinch bug, mole crickets, and white grubs (such as the Japanese beetle, *Popillia japonica* Newman) [7]. Because many nontarget arthropods, including beneficial arthropods, dwell in the turfgrass [8,9], it is critical to understand how these insecticides impact them to develop effective and sustainable pest management strategies and accordingly refine the integrated pest management (IPM) program.

The tiger beetle, *Megacephala carolina carolina* L. (Coleoptera: Carabidae), is common in turfgrass and ornamental landscapes in central Georgia, as they were regularly captured in pitfall traps [10,11]. The holes inhabited by *M. carolina carolina* larvae are regularly observed along bare-ground patches within turfgrass lawns and along the edges of the lawns (SVJ unpublished). *Megacephala carolina carolina* is a promising predator against multiple insect pests, such as *P. bicincta* adults [4] and those in the larval stages of *S. frugiperda* [12]. When *M. carolina carolina* adults were provided with prey in a choice assay between adult *P. bicincta* and late instars of *S. frugiperda*, they preferred the larvae of *S. frugiperda* over adult *P. bicincta* [12]. Therefore, *M. carolina carolina* could play an important role as a biological control agent in turfgrass systems, and their habitat in and around turfgrass systems should be protected to conserve *M. carolina carolina* populations.

**Figure 1:**
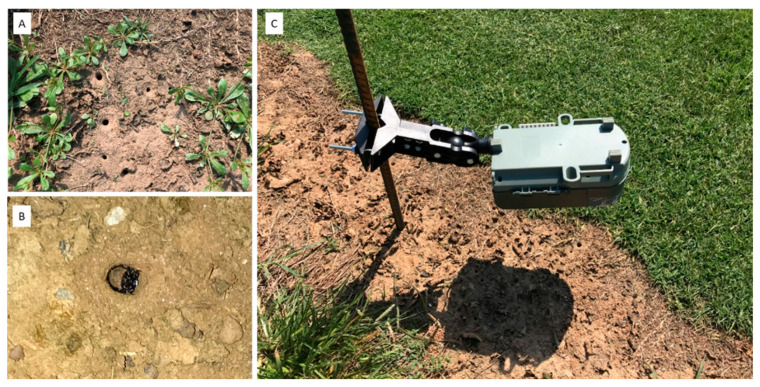
(**A**) Tunnels (visible as circular holes) on the soil surface with those in the larval stages of *M. carolina carolina*, (**B**) larva of *M. carolina carolina* at hunting position with head and thorax on the surface of the tunnel, and (**C**) bird-view camera deployed over the tunnels to capture the activities of larvae with time-lapse photos.

Similar to most other tiger beetles, *M. carolina carolina* adults have cursorial legs with shorter trochanters. *M. carolina carolina* adults are flightless and mostly nocturnal in habit, and adults hide under turfgrass patches during daytime. The females oviposit eggs in the soil about 1 cm deep along grassless bare-ground areas or edges of turfgrass lawns [13]. The developing larvae excavate tunnels in the soil, and the entrances of the tunnels appear as rounded holes (Figure 1A). When at hunting posture, the head and thorax of the *M. carolina carolina* larvae are seamlessly flushed along the hole and surface of the soil (Figure 1B). The head and thorax of larvae are heavily sclerotized and ventrally bent by 45° [13]. Additionally, the medial hooks on the fifth abdominal segment support the larvae to stand along the tunnel near the hole and hunt for prey. They ambush any approaching prey via a swift leaning movement and snatch the prey [13]. The diameter and depth of the tunnels increase as larvae develop into later instars inside the tunnels. They remain in tunnels unless threatened by other predators or the tunnels are inundated with rainwater. Adults and larvae have a pair of long sickle-shaped mandibles used to devour the prey.

Insecticides, including pyrethroids, could induce nontarget effects on coleopteran and beneficial arthropods, including lady beetles [14,15,16,17]. Exposure to pyrethroids can alter their normal behavior as they affect the voltage-sensitive sodium channels in the nervous system [18,19]. Similarly, imidacloprid can pose lethal and sub-lethal effects to predators, such as *Serangium japonicum* Chapin (Coleoptera: Coccinellidae) [20] and *Ceratomegilla undecimnotata* Schneider (Coleoptera: Coccinellidae) [21]. However, it is unclear how the predatory behavior of *M. carolina carolina* larvae and adults is affected when exposed to pyrethroids and neonicotinoids. Thus, the objective of the study was to determine the sub-lethal effects of bifenthrin (pyrethroid) and imidacloprid (neonicotinoid) residues on the predatory behavior of *M. carolina carolina* adults and larvae. This information could be incorporated into the IPM programs to manage key arthropod pests in turfgrass.

## 2. Materials and Methods

### 2.1. Study Site and General Methods

The experiments were conducted at the University of Georgia, Griffin campus, Griffin, GA, USA, in 2019 and 2020. The turfgrass area (~3317.5 m^2^) used for the experiment was located in the southeastern section of the 80,042.21 m^2^ open turfgrass field (Latitude: 33.260053; Longitude: −84.280958). The experimental plots were assigned on bare soil with limited turfgrass along the edges of bermudagrass (*Cynodon* spp.). The bermudagrass was mowed weekly at 2–4 cm height and irrigated daily for 30 min. However, the selected area of the turfgrass field was not exposed to insecticides during the growing season. The bare soil areas of bermudagrass were partially infested with weeds, such as southern crabgrass, Digitaria ciliaris (Retz.), and white clover, Trifolium repens L.

### 2.2. Larval Activity

The larval activity of *M. carolina carolina* on the surface of the circular holes was observed by deploying a bird-view camera (Pro WCT-00126; Wingscapes TimelapseCam, Calera, AL, USA). The camera was deployed at 15 cm (focal length) to focus on two tunnels (circular holes), and time-lapse images were recorded every 30 s for 24 h. The flash of the camera was triggered at 30 s intervals during the nighttime. A camera was assigned to a 1 m × 0.5 m plot area with larval colonization, and the area served as the experimental unit. The treatments were activity during (1) daytime and (2) nighttime. The treatments were replicated three times (with three cameras) with a completely randomized design. Daytime was from 4 PM to 8 PM, and nighttime was from 12 AM to 4 AM. The experiment was initiated on 23 September 2019. The mean daytime air temperature was ~30 °C, and the mean nighttime air temperature was ~21 °C in the turfgrass field. There were no rain events recorded during the experiment. The relative humidity was 60–90% and ~80–90% during daytime and nighttime, respectively.

### 2.3. Effect of Bifenthrin and Imidacloprid on Larval Activity

The experiments with bifenthrin and imidacloprid were conducted in 2019 and 2020, respectively. For bifenthrin, Talstar^®^ P Professional (7.9% bifenthrin FMC Corporation, Philadelphia, PA, USA) was used, whereas, for imidacloprid, Merit^®^ 2F (21.4% imidacloprid, Bayer Environmental Science, Research Triangle Park, NC, USA) was used. For each insecticide, the treatments were (1) 0.0×, (2) 0.25×, and (3) 1.0× of full label rate. The 0.0× was the nontreated control. The 0.25× treatment used 0.19731 L per ha and 0.29063 L per ha of Talstar (bifenthrin) and Merit (imidacloprid), respectively. The full label rates (1.0×) of Talstar and Merit were 0.78925 L per ha and 1.1625 L per ha, respectively. The water volume was 373.9 L per ha. The treatments were arranged in a randomized complete block design with four replications. The plot size was 1 m × 0.5 m. After preparing the insecticide solution, it was sprayed on the plot areas with at least two circular holes using a CO_2_-powered single boom (one nozzle) handheld sprayer at 206.8 kPa (kilopascal). The TeeJet 8002VS (yellow-colored tip, TeeJet Technologies, Glendale Heights, IL, USA) tip (flat cone) was used for spraying insecticide treatments. Thus, the insecticide residues were uniformly deposited on the plots.

After insecticide applications, a bird-view camera was deployed in each plot (experimental unit), and time-lapse digital photos were captured every 30 s for 24 h. The focal length of the cameras was adjusted so that they focused on at least two circular holes (tunnel entrances) in each plot. When at the hunting position, the *M. carolina carolina* larvae stayed on the top of the tunnels. The frequency of larvae on the top of the two tunnels was quantified after evaluating every photo captured for 24 h. The percentage of events when they were on the top of the tunnel (hunting position) was then calculated. An event is defined as the presence or absence of *M. carolina carolina* larvae on the surface of the tunnel at every 30 s. The experiment was set up from ~2 PM on day 1 to ~4 PM on day 2. This experiment was repeated for each insecticide (bifenthrin and imidacloprid). The experiments with bifenthrin were conducted on 9 and 16 October 2019, whereas the experiments with imidacloprid were conducted on 22 and 29 September 2020. In 2019, the mean daytime air temperature was ~33 °C, and the mean nighttime air temperature was ~26 °C in the turfgrass field. In 2020, the mean daytime air temperature was ~31 °C, and the mean nighttime air temperature was ~22 °C in the turfgrass field. Rain events were not recorded during the experiments in both years.

### 2.4. Effect of Bifenthrin on Adult Mobility

In 2019, *M. carolina carolina* adults were collected from the turfgrass fields in the University of Georgia, Griffin campus, Griffin, Georgia. The beetles were active after dusk on warm and rainless nights. They were collected from 9 PM to 11:30 PM when it was completely dark. Headlamps were used to locate the beetles. Most of the beetles were found hiding near turfgrass patches. They were hand-picked and released into 6 L plastic containers (Pioneer Plastics, 6.1 L Round Plastic Container, model#289C, Dixon, KY, USA) with four layers of moist paper towel in the bottom of the containers. The plastic containers with beetles were transported to the entomology laboratory. Before experiments, the *M. carolina carolina* adults were sorted into females and males. The males of *M. carolina carolina* have reddish–brown tarsal pads on the forelegs, which are absent for females. The air temperature in the laboratory was ~21 °C when the experiments were conducted.

The mobility of *M. carolina carolina* adults was determined after exposing them to bifenthrin-treated and nontreated filter papers in a Petri dish assay. The treatments were (1) 0.0×, (2) 0.25×, (3) 0.5×, and (4) 1.0× of the full rate of Talstar. The 0.25× and 0.5× treatments used 0.19731 L per ha and 0.39463 L per ha of Talstar (bifenthrin) and Merit (imidacloprid), respectively. The full label rate (1.0×) of Talstar^®^ P Professional (7.9% bifenthrin) was 0.78925 L per ha. Tap water was used at 373.9 L per ha. The insecticide solutions for various treatments were prepared ~1 h before the assay. The filter papers (Whatman#1, 90 mm diameter, Buckinghamshire, UK) were dipped in the insecticide treatment solutions for 6 s and dried in a hood for 30 min. After 30 min, the filter papers were placed on the lids of 9 cm disposable plastic Petri dishes, and *M. carolina carolina* adults were individually introduced into the Petri dishes. The Petri dishes with beetles were then placed on the LED light pad (Huion, Model#L4S, DC 5V [1W], Shenzhen, China). The walking behavior of adult *M. carolina carolina* on the LED light pad with the full light setting on was videotaped using an Ethernet camera (acA1300-60 gm, Basler, Inc., Exton, PA, USA) for 15 min. Total distance and average velocity moved by the adults were quantified using Noldus EthoVision XT software (Version 11.5, Noldus Information Technologies, Wageningen, The Netherlands). Lighted pads were used so that the detection of adult beetles could be improved using the EthoVision XT software. Six *M. carolina carolina* adults (females or males) were individually assayed using EthoVision XT software at any given time. Assays were replicated 8 and 12 times for females and males per treatment, respectively. These experiments were conducted from 3 August to 16 October 2020. Assays with adults were not conducted for imidacloprid because no apparent behavioral effects were observed with those in the larval stages.

### 2.5. Statistical Analyses

The data were analyzed using SAS software, Version 9.3 [22]. For larvae, the proportional data related to larval activity on the surface of two tunnels were subjected to analysis of variance (ANOVA) using the PROC TTEST procedure in SAS after arcsine square root transformation. The data evaluated during daytime and nightline were the treatments, with three replications. Similarly, the proportional data of larval activity to determine the effect of bifenthrin and imidacloprid were arcsine square root transformed. They were subjected to ANOVA using the PROC GLM procedure in SAS. The treatments were insecticides (and their rates) and were replicated four times. The repeated experiments were not combined and were analyzed separately. The means were separated using the Tukey HSD test (α = 0.05). The means and standard errors were calculated using the PROC MEANS procedure on nontransformed data in SAS.

For adults, the total distance and velocity data of adult movement were subjected to one-way ANOVA according to sex using a generalized linear model using the PROC GLIMMIX procedure in SAS. The models had a log-link function with a negative binomial distribution. The method used was Laplace. The treatment (total distance or velocity) was the fixed effect, and replication was the random effect in the models. The means were separated using the Tukey–Kramer least square test (α = 0.05). The means and standard errors were calculated using the PROC MEANS procedure on data in SAS.

## 3. Results

### 3.1. Larval Hunting Behavior

**Figure 2:**
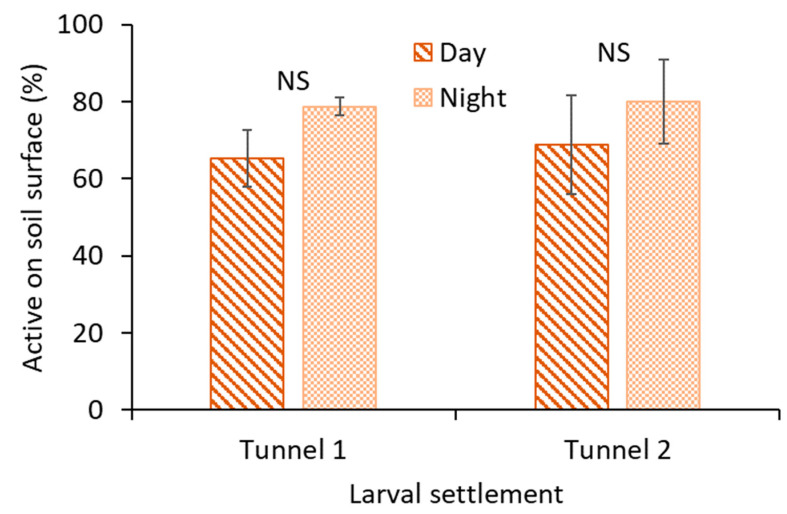
Mean (± SE) percentage of larvae observed at hunting position in tunnel 1 and tunnel 2 when observed during daytime and nighttime. No insecticide was applied. The means were separated using the Tukey HSD test (α = 0.05). NS = not significantly different.

A total of 240 photos were evaluated for each replication. There were no significant differences in larval hunting behavior between day and nighttime hours for tunnel 1 (t_4_ = −1.0; *p* = 0.358) and tunnel 2 (t_4_ = −1.1; *p* = 0.323; Figure 2).

### 3.2. Effect of Bifenthrin on Larval Activity

**Figure 3:**
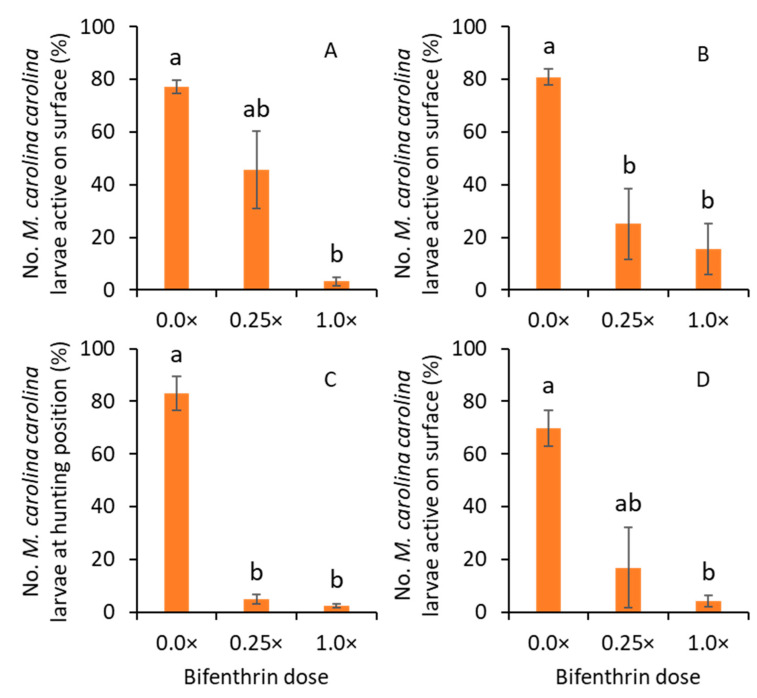
Mean (± SE) percentage of larvae observed at hunting position in (**A**) tunnel 1 and (**B**) tunnel 2 in trial 1, and (**C**) tunnel 1 and (**D**) tunnel 2 in trial 2 when bifenthrin was sprayed on the turfgrass field. Same letters among bars within each figure indicate being not significantly different at α = 0.05 (Tukey HSD test).

For trial 1, tunnel 1, significantly lower percentages of larvae were at the hunting position for 1.0× than for the 0.0× treatments (F_2, 5_ = 10.7; *p* = 0.014; Figure 3A). For tunnel 2, both 1.0× and 0.25× treatments had significantly lower percentages of larvae at the hunting position than for the 0.0× treatment (F_2, 5_ = 24.2; *p* = 0.003; Figure 3B). For trial 2, tunnel 1, significantly lower percentages of larvae were observed for the 1.0× and 0.25× treatments than for the 0.0× treatment (F_2, 4_ = 118.5; *p* < 0.001; Figure 3C). In tunnel 2, the 1.0× treatment significantly reduced the larvae at the hunting position compared with the 0.0× treatment (F_2, 4_ = 10.4; *p* = 0.026; Figure 3D).

### 3.3. Effect of Imidacloprid on Larval Activity

**Figure 4:**
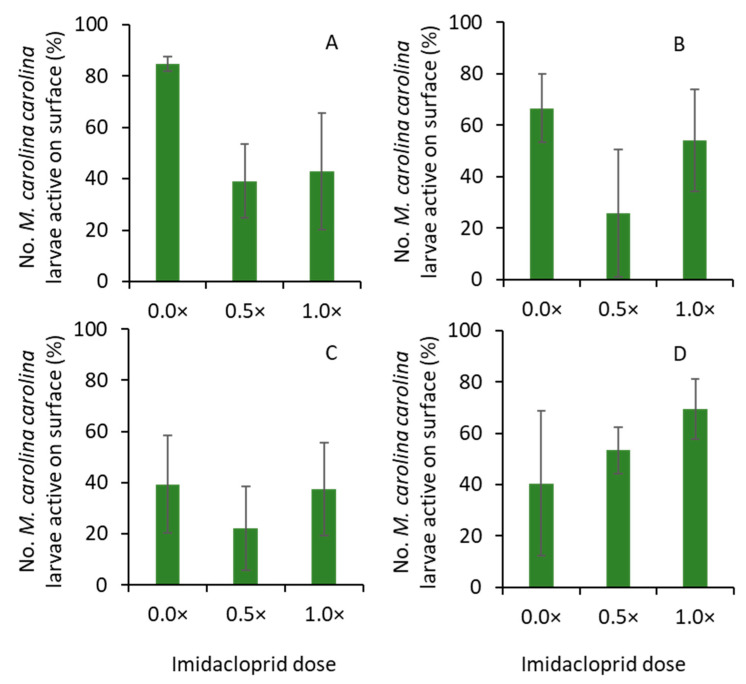
Mean (± SE) percentage of larvae observed at the hunting position in (**A**) tunnel 1 and (**B**) tunnel 2 in trial 1, and (**C**) tunnel 1 and (**D**) tunnel 2 in trial 2 when imidacloprid doses were sprayed on the turfgrass field. Significant differences among treatments were tested at α = 0.05 (Tukey HSD test). Where no significant differences were observed, no letters are included.

When treatment effects were analyzed, there were no significant differences among treatments for trial 1, tunnel 1 (F_2, 4_ = 3.1; *p* = 0.156; Figure 4A), or tunnel 2 (F_2, 5_ = 0.8; *p* = 0.551; Figure 4B). Similarly, for trial 2, the percentages of larvae at the hunting position were not significantly different for tunnel 1 (F_2, 5_ = 0.5; *p* = 0.629; Figure 4C), or tunnel 2 (F_2, 3_ = 0.1; *p* = 0.903; Figure 4D).

### 3.4. Effect of Bifenthrin on Adult Mobility

**Figure 5:**
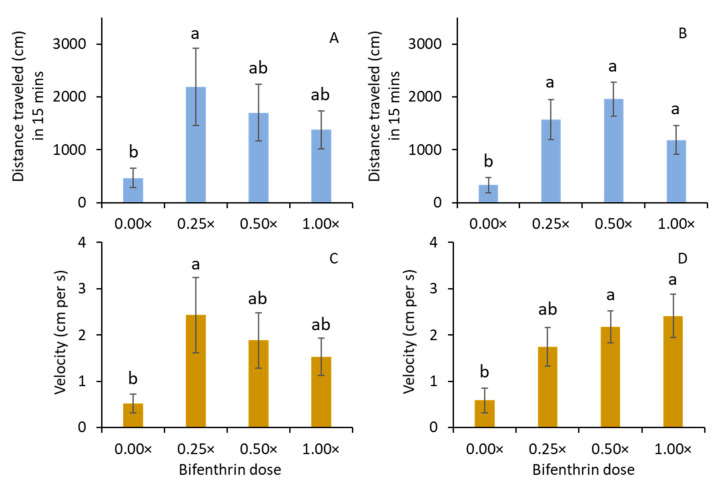
Mean (± SE) distance traveled by *M. carolina carolina* adult (**A**) females, (**B**) males, and velocity of (**C**) females and (**D**) males in Petri dish assay when bifenthrin doses were sprayed on filter paper in the laboratory. The same letters indicated among bars within each figure are not significantly different at α = 0.05 (Tukey–Kramer test). Where no significant differences were observed, no letters are included.

For females, the total distance traveled was significantly greater for the 0.25× treatment than for the 0.00× treatment (F_3, 29_ = 4.7; *p* = 0.011; Figure 5A). There were no significant differences among 0.25×, 0.5×, and 1.0× treatments or among 0.0×, 0.5×, and 1.0× treatments. For males, the total distance traveled was significantly greater for the 0.25×, 0.5×, and 1.0× treatments than for the 0.0× treatment (F_3, 21_ = 7.9; *p* < 0.001; Figure 5B). The velocity of female movement was significantly greater for the 0.25× treatment than for the 0.00× treatment (F_3, 21_ = 3.4; *p* = 0.037; Figure 5C). For males, the velocity of movement was significantly greater for the 0.5× and 1.0× treatments than for the 0.00× treatment (F_3, 29_ = 4.0; *p* = 0.017; Figure 5D). There were no significant differences in velocity among 0.25×, 0.5×, and 1.0× or between 0.0× and 0.25× treatments.

## 4. Discussion

The time-lapse photos show that the *M. carolina carolina* larvae were at the hunting position during the day and night. This suggests that *M. carolina carolina* larvae are persistent in seeking prey resources in the turfgrass ecosystem. Food can be a limiting factor for *M. carolina carolina* larvae in most landscapes, and they spend more time in the hunting position seeking food to meet their developmental needs [23]. Thus, if *M. carolina carolina* larvae were unable to hunt, it could severely cost them in an already prey-limited habitat. When lower doses of bifenthrin were applied to the bare ground areas, where larvae of *M. carolina carolina* were noted, their hunting behavior was impaired compared to nontreated areas. Similarly, the movement of *M. carolina carolina* adults increased after exposure to sub-lethal doses of bifenthrin. These results were consistent with previous studies, where pyrethroids, including bifenthrin, induced sub-lethal effects, such as changes in movement or grooming behaviors after exposure in many arthropods [18,24,25]. However, imidacloprid did not affect the hunting behavior of *M. carolina carolina* larvae in the current study. Previously, sub-lethal doses of imidacloprid induced changes to certain behaviors in beneficial arthropods [26] while not affecting other behaviors [27]. This suggests that the behavioral responses of *M. carolina carolina* larvae can vary by the type of active ingredient used and the type of behavior under investigation. Thus, turfgrass managers should be cautious when applying certain insecticides, especially bifenthrin, to avoid potential exposure to the larvae of *M. carolina carolina* colonized in turfgrass.

It is unclear as to why bifenthrin residues induced hyperactivity in *M. carolina carolina* larvae, whereas imidacloprid residues did not induce any detectable changes to larval activity. It was observed that the larvae of *M. carolina carolina* crawl down inside the tunnel when an object, such as spray equipment or a human, approaches the tunnels. *M. carolina carolina* larvae possibly crawled down inside the tunnel just before the insecticide was applied and avoided direct exposure. However, when the *M. carolina carolina* larvae returned to the hunting position, they might have been exposed to the bifenthrin residues deposited on the soil surface via contact with their appendages, such as legs or antennae. The absence of *M. carolina carolina* larvae at the hole of the tunnels was possibly caused by a hyperactivity reaction after exposure. It is unclear whether the larvae recovered or died after exposure because they were not monitored beyond 24 h. The sensitivity of *M. carolina carolina* larvae to imidacloprid residue could be weak and did not result in any quantifiable response. Kunkel et al. (2001) showed that the adult ground beetle, *Harpalus pennsylvanicus* DeGeer (Coleoptera: Carabidae), did not differentiate between the imidacloprid-treated and nontreated surfaces. However, imidacloprid was very toxic to the larvae of *H. convergens,* with direct exposure causing 100% mortality [27].

The *M. carolina carolina* adults walked a further distance at a greater velocity when exposed to lower doses of bifenthrin than the nontreated control. This result is consistent with previous studies where the mobility of coleopteran predators was reportedly increased after exposure to pyrethroid residues [28,29]. For example, the grooming and walking behaviors of the ladybird beetle, *Coccinella septempunctata* L. (Coleoptera: Coccinellidae), increased after exposure to deltamethrin spray on plants [28]. Similarly, video-tracking experiments suggest that walking distance, velocity, and movement time of the ground beetle, *Scarites quadriceps* Chaudior (Coleoptera: Carabidae), increased after exposure to lambda-cyhalothrin or tefluthrin [29]. This enhanced mobility of arthropods was likely caused by hyperactivity after exposure to pyrethroids. Wiles and Jepson (1994) have implicated that pyrethroids can irritate the cuticle of insects. It is unclear whether the hyperactivity in *M. carolina carolina* adults would affect or enhance prey-finding behavior in turfgrass. Although *M. carolina carolina* adults are cursorial hunters [13], they typically wait along the edges of turfgrass patches to locate the prey. Once they locate the prey, they chase the prey and grab it using their mandibles. This normal hunting behavior may be altered after exposure to bifenthrin residues and will likely result in fewer prey captures. Additionally, the hyperactive mobility of pyrethroid-exposed adults may increase the risk of being a victim of predation [24,26] or parasitism [30] upon gaining attention from other predators and parasitoids. In contrast, other studies have shown that the movement of adult honey bees [18] and the striped lynx spider, *Oxyopes salticus* Hentz [25], was reduced after exposure to pyrethroids.

## 5. Conclusions

The results show that the larvae of *M. carolina carolina* were more sensitive to sub-lethal doses of bifenthrin than imidacloprid as they spent less time hunting for prey by sitting at the entrance of the tunnel. The mobility of adult *M. carolina carolina* was affected by sub-lethal doses of bifenthrin in the laboratory assays. Moreover, the larvae of *M. carolina carolina* were active during the daytime and nighttime hours. The landscape maintenance and installation companies serving residential lawns and business properties, golf course superintendents, and public park and installation managers must carefully scout for the presence of tiger beetle colonies inside and along the edges of turfgrass fields. If patches of tunnels are detected, they should avoid spraying those colonies with bifenthrin or other pyrethroids. Bifenthrin is commonly used to manage lepidopteran pests, ants, etc. Results suggest that not all insecticides induce nontarget effects equally to arthropods. The sub-lethal doses of imidacloprid did not alter larval hunting behavior, as imidacloprid is widely used against many insect pests, including white grubs, in various landscapes. More research is warranted to determine the longer-term effects of these insecticides on the hunting behavior and survival of *M. carolina carolina* larvae. Similarly, future studies should focus on *M. carolina carolina* adults to understand how they adapt to environments where insecticides are routinely applied to manage other key insect pests. This information will ultimately help to conserve *M. carolina carolina* in various turfgrass landscapes so that we benefit from the biological control services they provide in managing key insect pests. The information generated from the current study will refine the IPM programs in golf courses, athletic fields, and public and residential lawns.

## Data Availability

Data will be provided upon request.
